# Amplifying the voices of disabled people: a model for better community engagement in eye health

**Published:** 2022-09-20

**Authors:** Syeda Munazza Gillani, Leena Ahmed

**Affiliations:** Country Director: Sightsavers Pakistan.; Associate Programme Manager: Sightsavers Pakistan.

**A project based on partnerships with disabled people in Pakistan offers a model for improving eye health delivery to the most marginalised members of society.**

According to the World Health Organization World Report on Disability, 2011, there is a strong relationship between disability and poverty: disability can increase the risk of poverty, and poverty can increase the risk of disability,^1^ with access to health care being an important factor.

**Figure:**
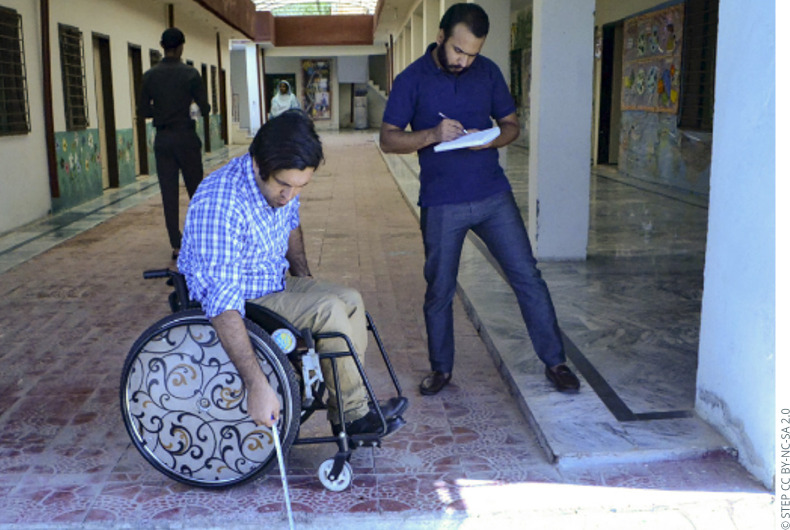
A member of a local organisation of people with disabilities (OPD) evaluates how accessible a health facility is for people with disabilities. pakistan

In Pakistan, high levels of discrimination and social exclusion affect individuals with disabilities and their families,^2^ making it difficult for them to afford eye care and get access to the eye care they need.

“In Pakistan, high levels of discrimination and social exclusion affect individuals with disabilities and their families, making it difficult for them to afford eye care and get access to the eye care they need.”

To address this challenge, and after testing a similar model in India, Sightsavers set up an inclusive eye health programme in four districts in Pakistan in 2018. Inclusive eye health means ensuring that eye care services are set up in consultation with people with disabilities and their representative organisations. It also means ensuring that eye care services are accessible and welcoming for everyone. Services are then more accessible for all neglected groups, particularly women, children, and people with disabilities.

The project focused on two key areas:

- Generating demand for eye care among people with disabilities in the community
- Ensuring that services were accessible and inclusive, and that they met the community's eye care needs.

The project's main implementing partner was the charity hospital network Layton Rahmatulla Benevolent Trust (LRBT). To ensure equity and inclusion, we partnered with a national organisation of people with disabilities, known as Special Talent Exchange Programme (STEP). The project consisted of several stages:

1. Situational analysis & stakeholder mapping
2. Identifying barriers
3. Addressing provider-side barriers
4. Improving demand for eye care
5. Advocacy and policy change
6. Monitoring and evaluation.

## 1. Situational analysis

For each of the four districts, we collected baseline data on the level of access of people with disabilities, identified areas where poverty and disability are more prevalent, and collected sociodemographic information.

We also mapped and analysed who was involved in, or could influence, the success of the programme, and what their level and area of influence might be. These included community leaders, religious scholars, and local political figures who could influence and advocate for improved uptake of, and access to, health care for local communities in general and disabled people in particular.

## 2. Identifying barriers

We engaged with local organisations of people with disabilities and asked them to help us to identify any barriers in the way of inclusive eye health.

In addition to provider-side barriers (see Table 1), we also identified a lack of demand for eye care in the community. This was due to a lack of awareness in the community about the eye care needs of people with disabilities, a lack of awareness in the community and among people with disabilities themselves about the services that are available, as well as unhelpful attitudes towards people with disabilities.

**Table 1:** Provider-side barriers and their solutions.

**Barriers identified**	**Solutions**
**Attitudes of staff members**	Sensitisation training for hospital staff on disability and gender rights
**Physical infrastructure and organisational development**	Inviting people with disabilities to assess how accessible and disability-friendly health facilities are (known as an ‘accessibility audit’) Retrofitting facilities with disability-friendly and accessible infrastructure Improving communication between eye care and other health or rehabilitation services to ensure better referrals and follow-up
**Communication barriers**	Providing accessible communication material, e.g., written information in Braille or audio material for people who are blind or visually impaired, and sign language interpretation for people who are hearing impaired
**Transportation barriers**	Providing dedicated outreach camps closer to the communities or offering free or subsidised transportation

## 3. Addressing provider-side barriers

In our partnership with organisations of people with disabilities, we learned that bringing services nearer to the communities through eye care screening camps and making services available at the primary health care level is a great motivator for marginalised people who otherwise cannot access eye health services. However, once we create demand for eye care services, it is equally important that services are accessible, inclusive, and equitable.

Local organisations of people with disabilities were invited to carry out disability access audits of eye hospitals and recommending essential infrastructural modifications. With the local self-help groups (see below), they actively supported disability awareness raising – via sensitisation training – for hospital staff. They also helped us to establish a database of persons with disabilities so we could monitor who was receiving care.

## 4. Improving demand for eye care

To address the lack of demand, we needed to improve everyone's understanding of the eye care needs of disabled people and the services that are available for them. We also had to challenge negative attitudes towards eye care for disabled people amongst community members, leaders, and decision makers.

### Setting up self-help groups

As a first step in addressing lack of demand, we worked with the elected union councils (also known as village councils) in each of the four districts to create self-help groups that included people with disabilities.

In Pakistan, self-help groups are relatively common. They are informal groups of local people (such as teachers, political and religious leaders, and representatives from women's empowerment focused-groups and others) who come together to discuss issues and problems they have in common. Self-help groups, as the name implies, look for solutions on a self-help basis but they do sometimes seek help from local government institutions, such as union council offices. The groups also promote social cohesiveness through local cultural events and gatherings, street theatres to promote a collective approach towards social issues, and health and hygiene sessions.

**Figure:**
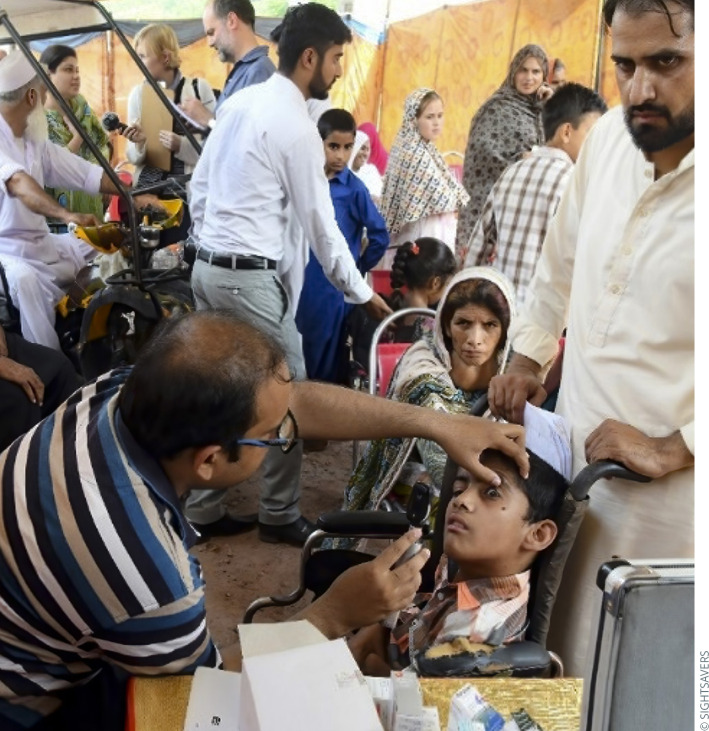
An eye screening camp in the community for people with disabilities that was planned with the support of a local organisation of people with disabilities (OPD) partner. pakistan

Initially, Sightsavers' project team supported the organisation of these self-help groups and encouraged the inclusion of people with disabilities. A total of 57 self-help groups were created in the four districts, and each included an average of 10–12 people with disabilities. The self-help groups worked closely with LRBT hospital staff members to arrange accessible and dedicated eye care outreach screening camps for people with disabilities and other marginalised groups who otherwise could not come to the hospital themselves. 

### District coordination committees

District coordination committees were organised in each of the four districts to coordinate the work of the self-help groups and to better link them with local government structures. Members of local organisations of people with disabilities were also made members of the district coordination committees in order to amplify their voices in local decision-making processes.

Together, the district coordination committees helped more than 100 people with disabilities to get their disability certificates and special national identification cards. Similarly, the medical assessment of people with disabilities (which are needed before disability certificates can be issued) is a very lengthy and cumbersome process that requires a visit to the main hospital in each district, usually very far away. However, with the support of the district coordination committees, eye health screening camps were arranged in the villages nearer to the communities.

### Creating social behaviour change programmes to generate demand

The self-help groups and local organisations of people with disabilities took active part in the design and delivery of a social behaviour change campaign to improve the uptake of eye care services by all, especially people with disabilities. This involved:

- disseminating printed eye health information and educational materials
- carrying out communal awareness raising activities such as street theatres
- people with disabilities acting as role models and speaking at communal forums during eye care outreach camps (see panel).

## 5. Advocacy and policy change

Local organisations of people with disabilities supported advocacy by engaging community leaders in fighting the stigma of disability, supported by the project and other data, research, and best practice guidelines. It helped that the project was planned in close alignment with the existing national health plan (National Health Vision of Pakistan 2016-2025),^2^ which endeavours to develop a well-informed and gender-responsive national plan to tackle several health challenges, maintaining and prioritising universal health coverage as its ultimate goal.

“So far, the qualitative feedback and the evaluation report showed that the project has made the partner hospitals more accessible and welcoming for people with disabilities.”

**People with disabilities as role models**

Unhelpful attitudes towards people with disabilities – as somehow being less worthy of health care and eye care – is a key barrier when it comes to getting access to eye care. To break down social taboos and stigma around disability, disabled people were invited to address the community members attending outreach eye camps.

At one of the outreach camps, a female leader of an organisation of people with disabilities spoke as follows: “I live with a physical disability, but it did not stop me from getting education. I completed my undergraduate degree and am now applying for work and planning to get married. I am also the secretary of a self-help group where I lead social work, informing the people about eye camps and raising their awareness about importance of eye care treatment. I am working closely with a disability rights awareness team supported by the organisation where I work as a volunteer.”

## 6. Monitoring and evaluation

The team collected data separately for people with disabilities, and by gender (known as gender- and disability-aggregated data) to measure inequality in access and to monitor the progress of the project.

## Outcomes and next steps

The project has created an inclusive eye health model that can be scaled up and replicated, especially in government health facilities. As a result, Pakistan's national eye care plan now has strong elements of equity and inclusion embedded within it, forming an integral part of the design.

Quantitative published data from the project is not yet available. However, so far, the qualitative feedback and the evaluation report showed that the project has made the partner hospitals more accessible and welcoming for people with disabilities. They also showed that access to eye care has improved, thanks to community outreach screening eye camps that have boosted the uptake of eye care services in marginalised communities, especially people with disabilities. It is anticipated that self-help groups will continue to be part of the local organisational structures in the community, eventually extending beyond eye health to support broader thinking and consultation on a range of issues.

The effective inclusion of people with disabilities in eye health services will pave the way for people with disabilities to demand the same in other areas of health. We hope that this will ultimately lead to achieving universal health coverage in Pakistan and ensuring that people with disabilities are not left behind.
